# Cedrol restricts the growth of colorectal cancer *in vitro* and* in vivo* by inducing cell cycle arrest and caspase-dependent apoptotic cell death

**DOI:** 10.7150/ijms.77719

**Published:** 2022-10-31

**Authors:** Ju-Huei Chien, Kai-Fu Chang, Shan-Chih Lee, Chien-Ju Lee, Yi-Ting Chen, Hung-Chih Lai, Yin-Che Lu, Nu-Man Tsai

**Affiliations:** 1 Department of Research, Taichung Tzu-Chi Hospital, Buddhist Tzu-Chi Medical Foundation, Taichung 42743, Taiwan, R.O.C.; 2 Department of Medical Laboratory Science and Biotechnology, Central Taiwan University of Science and Technology, Taichung 40601, Taiwan, R.O.C.; 3 Department of Medical Laboratory and Biotechnology, Chung Shan Medical University, Taichung 40201, Taiwan, R.O.C.; 4 Department of Medical Imaging and Radiological Sciences, Chung Shan Medical University, Taichung 40201, Taiwan, R.O.C.; 5 Department of Medical Imaging, Chung Shan Medical University Hospital, Taichung 40201, Taiwan, R.O.C.; 6 Division of Hematology and Oncology, Department of Internal Medicine, Shin-Kong Wu Ho-Su Memorial Hospital, Taipei 11101, Taiwan, R.O.C.; 7 Institute of Pharmacology, National Taiwan University, Taipei 10617, Taiwan, R.O.C.; 8 Division of Hematology‑Oncology, Ditmanson Medical Foundation Chia-Yi Christian Hospital, Chia-Yi 60002, Taiwan, R.O.C.; 9 Min-Hwei Junior College of Health Care Management, Tainan 73658, Taiwan, R.O.C.; 10 Clinical Laboratory, Chung Shan Medical University Hospital, Taichung 40201, Taiwan, R.O.C.; 11 Department of Life-and-Death Studies, Nanhua University, Chiayi 62249, Taiwan, R.O.C.

**Keywords:** Cedrol, colorectal cancer, cell cycle arrest, apoptosis, synergistic effect

## Abstract

**Background:** Cedrol is a natural sesquiterpene alcohol found in *Cedrus atlantica*, which has been proven to have a broad spectrum of biological activities, such as antimicrobial, anti-inflammatory, analgesic, anxiolytic, and anti-cancer effects. However, the underlying anticancer mechanisms and *in vivo* inhibitory effects of cedrol on colorectal cancer (CRC) have not been elucidated. In the present study, we investigated the anti-CRC potential of cedrol using *in vitro* and *in vivo* models.

**Methods:** The effects of cedrol on cell viability, cell cycle progression, and apoptosis of HT-29 and CT-26 cells were detected by MTT, flow cytometry, and TUNEL assays. Western blotting was used to measure protein expression for molecular signaling analyses.

**Results:** Cedrol inhibited HT-29 and CT-26 cell proliferation in a time- and dose-dependent manner, with IC_50_ values of 138.91 and 92.46 µM, respectively. Furthermore, cedrol induced cell cycle arrest at the G0/G1 phase by regulating the expression of cell cycle regulators, such as CDK4 and cyclin D1, and triggered apoptosis through extrinsic (FasL/caspase-8) and intrinsic (Bax/caspase-9) pathways. In addition, cedrol in combination with the clinical drug 5-fluorouracil exhibited synergistic inhibitory effects on CRC cell growth. Importantly, cedrol treatment suppressed the progression of CRC and improved the survival rate of animals at a well-tolerated dose.

**Conclusion:** These results suggest that cedrol has an anti-cancer potential via induction of cell cycle arrest and apoptosis, and it could be considered as an effective agent for CRC therapy.

## Introduction

According to the World Health Organization (WHO), cancer was the second leading cause of death worldwide in 2020, being responsible for nearly 10 million deaths. Colorectal cancer (CRC) is a serious malignant disease and ranks as the third most common morbidity, accounting for approximately 10% of all new cancer cases and 9.4% of all cancer-related deaths, according to the global cancer statistics in 2020 1. CRC is widespread, especially in economically developed countries, which is attributed to lifestyle changes, including modern eating habits, transfer of aging/sex population, increase in smoking incidence, lack of exercise, and overweight and obesity 2. Current strategies for CRC treatment include surgery, chemotherapy, radiotherapy, targeted therapy, and immunotherapy 3. However, at the time of diagnosis, most patients have serious symptoms such as intestinal obstruction and bleeding, leading to the development of metastatic or advanced cancer (stages III and IV) 4. Systemic treatment with chemotherapy is recommended before or after surgery to reduce the risk of tumor recurrence and metastasis and improve the survival rate of patients with CRC 5. The most widely used chemotherapeutic drugs for CRC include 5-fluorouracil (5-FU), irinotecan, and oxaliplatin 3. 5-FU, a cycle-specific antimetabolite, is a first-line drug for CRC treatment and prevents the synthesis of DNA and RNA in cancer cells by significantly reducing thymidine, resulting in growth inhibition and cell death 6. However, the response rate to 5-FU in patients with advanced CRC is limited to 10-15%, and approximately 50% of patients with metastatic CRC exhibit resistance to 5-FU-based treatment 7-9. The resistance and side effects of 5-FU (e.g., leukopenia, anemia, and diarrhea) restrict the effectiveness of CRC treatment and reduce quality of life 10, 11. Therefore, the development of novel agents and strategies for the treatment of CRC is important.

Cancer has been a major health problem for decades, and it is still considered a serious cause of mortality worldwide. Early cancer treatment strategies focused on DNA synthesis and cell replication with non-selective toxicity, resulting in severe side effects 12, 13. Thus, the development of effective and safe anticancer drugs with different selectivities between cancer and normal cells is still being actively explored 14. Recent studies have reported that targeting specific biomarkers required for the regulation of cell division or apoptosis selectively affects the growth or supporting environment of cancer cells, exhibiting minimal influence on normal cells 15. During cell cycle progression, cyclin/cyclin-dependent kinases (CDK) form a family of heterodimeric kinases and play key roles in controlling cell division by regulating the transition in different cell cycle phases 16. Cyclin D1/CDK4 complex regulates the transition of the G_1_/S stage and initiates DNA synthesis, while CDK inhibitor p21 suppresses it 17. In addition, cell cycle progression affects apoptosis 18. Apoptosis is a major form of programmed cell death and is classified as either intrinsic or extrinsic based on its molecular mechanism 19. It is related to the regulation of the caspase cascade and has specific morphological features such as increasing the population of cells in the sub-G1 phase, condensation of chromatin, fragmentation of DNA, and formation of apoptotic bodies 20. The development of compounds that inhibit the cell cycle and induce apoptosis may be a promising approach to identify potential drugs for cancer therapy.

Natural products have been used to treat various diseases. Recently, there has been a growing interest in using natural substances derived from medicinal plants as potential anti-cancer agents or adjuvants with high efficacy and low toxicity 21. Cedrol, a type of naturally occurring sesquiterpene alcohol, is widely distributed throughout the plant kingdom and is particularly abundant in conifers (e.g., *Cedrus atlantica* and *Juniperus virginiana*) 22, 23. In previous studies, cedrol has been reported to exhibit various biological activities, such as antimicrobial, anti-inflammatory, hair growth-promoting, analgesic, and anxiolytic activities 24-28. Cedrol-induced autophagy and apoptosis in non-small cell lung cancer cells suppressed the growth of glioblastoma by enhancing DNA damage and attenuating drug resistance and chemosensitized cancer cells through the destabilization of plasma membrane lipid raft 29-32. However, little is known about the mechanisms underlying the growth inhibition and apoptosis associated with the anticancer effect of cedrol in CRC. The purpose of the current study was to investigate the inhibitory effects of cedrol on the progression of CRC using *in vitro* and *in vivo* models and discuss the mechanisms involved in the inhibition of cell proliferation and induction of apoptosis.

## Materials and Methods

### Compounds and Reagents

Cedrol, purity more than 98% determined by GC analysis, was purchased from Tokyo Chemical Industry Co., Ltd. (Tokyo, Japan), and 5-FU, dimethyl sulfoxide (DMSO), 3-(4,5-dimethylthiazol-2-yl)-2,5-diphenyltetrazolium bromide (MTT), propidium iodide (PI), and RNase A were purchased from Sigma-Aldrich (St. Louis, MO, USA). Primary antibodies for various proteins, including p-p53, p21, p-Rb, PCNA, CDK4, cyclin D1, CDK2, cyclin A, cyclin B1, and FasL, were obtained from Santa Cruz Biotechnology (CA, USA). Primary antibodies against caspase-8, Bax, caspase-9 caspase-3, and β-actin were purchased from iReal Biotechnology (Hsinchu, Taiwan). Cedrol and 5-FU were dissolved in DMSO to a final concentration of 0.5%. An equal volume of DMSO was added as a control at a final concentration of 0.5% in the medium.

### Cell Culture

HT-29 (human colorectal adenocarcinoma) cells were purchased from the American Type Culture Collection (ATCC, Rockville, MD, USA). CT-26 (mouse colorectal carcinoma), SVEC (mouse vascular endothelial), and MDCK (canine kidney epithelial) cells were purchased from the Bioresource Collection and Research Center (BCRC, Hsinchu, Taiwan). HT-29, SVEC, and MDCK cells were cultured in Dulbecco's modified Eagle's medium, and CT-26 cells were cultured in Roswell Park Memorial Institute Medium-1640 (RPMI) supplemented with 10% FBS, pyruvate, HEPSE, and streptomycin/penicillin in an incubator (5% CO_2_, 37 °C). All cell culture reagents were purchased from Gibco BRL (Life Technologies, Grand Island, NY, USA). A mutant (R273H) in TP53 exon 8 of HT-29 cells was detected using automatic nucleic acid extraction (AccuBioMed Co., Ltd., Taipei, Taiwan) and Femtopath Human TP53 Primer Set (HongJing Biotech, Taipei, Taiwan).

### Cell Viability Assay

The effect of cedrol on the viability of HT-29 and CT-26 cells was measured using an MTT assay. Cells at a density of 5 × 10^3^ cells/well were cultured in 96-well plates overnight and treated with cedrol (0, 14, 28, 56, 112, 225, and 450 μM) or 5-FU (0, 24, 48, 96, 192, and 384 μM) for 24, 48, and 72 h. The culture medium was replaced with MTT solution (400 μg/ml, Sigma-Aldrich) and incubated for 6-8 h at 37 °C. The formazan crystals were dissolved in 50 μL DMSO, and the intensity of absorbance at 560 nm was measured using a microplate reader (SpectraMax Plus 384, Molecular Devices, USA). Cell viability was expressed as the percentage of proliferation compared with that of the untreated group (100%). All measurements were performed in triplicate, and the experiments were repeated at least three times.

### Analysis of Cell Cycle Distribution

Flow cytometry was used to analyze the cell cycle distribution and cell death. HT-29 and CT-26 cells were seeded at 2 × 10^6^ cells in a 10-mm culture dish and then treated with different concentrations of cedrol for 0, 6, 12, 24, and 48 h. The treated cells were collected followed by incubation with the solution containing 40 µg/ml PI and 100 µg/ml RNase A in the dark overnight at 4 °C. Flow cytometric cell analysis was performed using FACScan (Veckton Dickinson, USA), and the results were analyzed using FlowJo software (Tree Star, San Carlos, USA).

### Evaluation of Apoptosis

Apoptosis was determined by immunofluorescence staining of terminal deoxynucleotidyl transferase dUTP nick end-labeling (TUNEL) using an *In situ* Cell Death Detection Kit (Roche, Mannheim, Germany). Treated cells were fixed with 4% paraformaldehyde, smeared, and dried on silane-coated slides. Cells or tissues on the slides were incubated with 3% H_2_O_2_ in methanol for 10 min and 0.1% Triton X-100 in 0.1% sodium citrate buffer for 2 min on ice. Subsequently, the samples were incubated with the TUNEL reaction mixture for 2 h, washed twice with PBS, and counterstained with PI staining. TUNEL results (green fluorescence) were observed using a fluorescence microscope (ZEISS AXioskop2) at 400× magnification.

### Western Blot Analysis

The protein expression in HT-29 cells after treatment with cedrol was evaluated by western blot analysis. Treated cells were collected and lysed in RIPA buffer (Bio Basic Inc., Canada) containing protease (Amresco Inc., USA) and phosphatase (Bionovas, Toronto, Canada) inhibitor cocktail on ice. After incubation for 30 min, the lysates were removed and the supernatant was collected by centrifugation at 14,000 × g for 30 min at 4 °C. Subsequently, the total protein concentration in each sample was measured using a BCA Protein Assay Kit (Pierce; Thermo Scientific, USA). Protein samples (20 µg/lane) were loaded, separated via 8-12.5% SDS-PAGE, and transferred onto 0.22 μm PVDF membranes (PALL Corporation, USA). The membranes were blocked using 5% skimmed milk in Tris-buffered saline containing 0.5% tween-20 (TBS-T, pH 7.4) for 30 min and incubated with primary antibodies diluted in 5% bovine serum albumin overnight at 4 °C. After washing thrice with TBS-T, the membranes were incubated with biotin-conjugated secondary antibodies (Santa Cruz, CA, USA) for 2 h and subsequently incubated with peroxidase-conjugated streptavidin (Jackson ImmunoResearch Inc., USA) for 1 h at room temperature. Detection was performed using an enhanced chemiluminescence reagent (ECL, T-Pro Biotechnology, Taipei, Taiwan) and chemiluminescence imaging analyzer (GE LAS-4000; GE Healthcare Life. Sciences, NJ, USA). The expression level of the protein band was analyzed using ImageJ software (version 1.8.0; National Institutes of Health, Bethesda, MD, USA) and standardized by comparison with β-actin expression.

### Drug Combination Treatment

Cells were digested, dispersed, and inoculated in 96-well plates at a density of 5×10^3^ cells/well, followed by overnight incubation in a 37 °C incubator. Thereafter, the cells were treated with cedrol (0, 45, 90, and 180 μM) combined with 15.4 μM 5-FU or 5-FU (0, 7.7, 15.4, and 30.8 μM) combined with 112 μM cedrol. After treatment for 48 h, cell viability was detected using the MTT assay. The effects of drug-drug interactions were determined based on the combination index (CI) theorem of Chou-Talalay to evaluate synergism (CI < 1), additive effect (CI = 1), and antagonism (CI > 1) using CompuSyn software (ComboSyn, Inc., Paramus, NJ, USA).

### *In vivo* Studies

The *in vivo* study was performed at Chung Shan Medical University (CSMU) and approved by the Institutional Animal Care and Use Committee (IACUC) of CSMU (Ethics Number: CSMU-IACUC-1543). BALB/c mice (10-12 weeks old, 20-23 g) were purchased from the National Laboratory Animal Center (Taipei, Taiwan) and maintained in temperature- and humidity-controlled rooms with a 12-h light/dark cycle. CT-26 cells (1 × 10^6^) were suspended in 100 μL PBS and subcutaneously injected into the right dorsal flank of the mice. After implantation for 7 days, the mice were randomly divided into vehicle (n = 5) and cedrol (n = 5) groups and received 100 μL of mineral oil and 150 mg/kg cedrol via subcutaneous injection (once every 2 days for 10 times). 5-FU (n = 3) was administered intraperitoneally at a dose of 25 mg/kg 3 times a week for 21 days as a positive control 33. The tumor size was measured using a caliper, and the volumes were calculated using the following formula: length × width × height × π/6 mm^3^. When the tumor volume exceeded 1500 mm^3^, the mice were euthanized using CO_2_. Tumor samples were collected, fixed with 4% formaldehyde, embedded in paraffin, sliced, and stained with H&E and TUNEL for histological examination 23.

### Statistical Analysis

Except for *in vivo* studies, all experiments were repeated at least three times. The results are expressed as the mean ± standard deviation (SD) (*in vitro*) or mean ± SEM (*in vivo*). Statistical analyses were performed using one-way analysis of variance for comparison between multiple groups or Student's t-test for comparison between two groups, performed using SPSS v16.0 software or Excel 2016 software. The Kaplan-Meier method was used to determine a significant difference in survival rate. *p* < 0.05 was considered statistically significant.

## Results

### Cedrol Inhibited the Growth of CRC Cells

**Figure 1:**
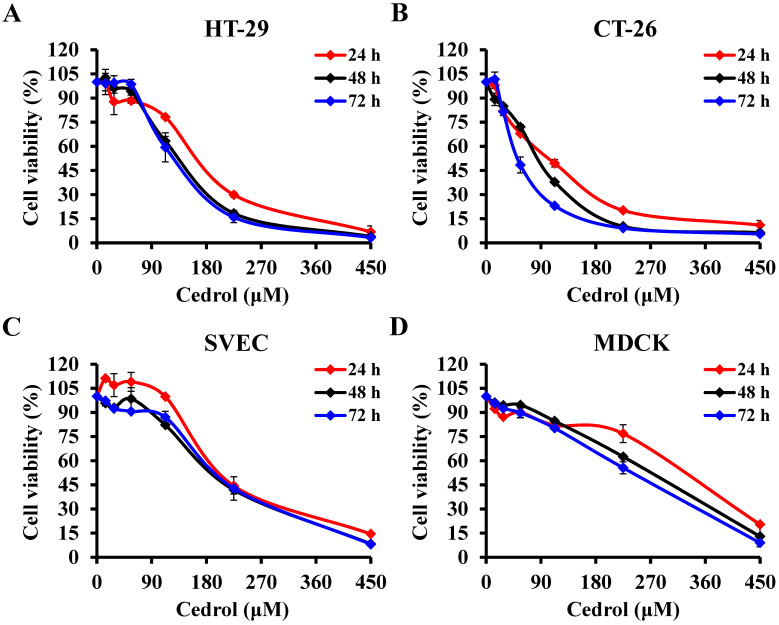
** The effect of cedrol on viability of CRC cells.** The percentage of cell viability in **(A)** HT-29, **(B)** CT-26, **(C)** SVEC, and **(D)** MDCK cells was measured using the MTT assay after cedrol treatment at the indicated concentrations (0-450 µM) for 24, 48, and 72 h. Results are presented as means ± SD from three independent experiments.

Cedrol is one of several components of the bark extract of *Cedrus atlantica* 23. To evaluate the *in vitro* anti-proliferative activity of cedrol on CRC cells, HT-29 and CT-26 cells were treated with cedrol (0-450 μM) for 24, 48, and 72 h. MTT assay results showed that cedrol significantly inhibited the growth of CRC cells in a dose- and time-dependent manner (Figure 1A and B). However, in comparison with CRC cells, cedrol had less of an effect on cell growth in normal cell lines, including SVEC mouse vascular endothelial cells and MDCK canine kidney epithelial cells (Figure 1C and D). As shown in Table 1, the IC_50_ values of cedrol in HT-29, CT-26, SVEC, and MDCK were 138.91 ±17.81, 92.46 ± 4.09, 202.19 ± 4.27, and 281.60 ± 5.17 μM respectively at 48 h of treatment. The values of selection index, a ratio between the IC_50_ values of the CRC and normal cell lines, were approximately 1.46 to 3.05 in cedrol treatment (Table 2), indicating that cedrol had the higher selection for CRC cells, whereas the effect was not found in the 5-FU treatment. These results suggest that cedrol had a more potent effect on the inhibition of CRC cell proliferation than on normal cells.

### Cedrol Induced Cell Cycle Arrest at G_0_/G_1_ Phase

**Figure 2:**
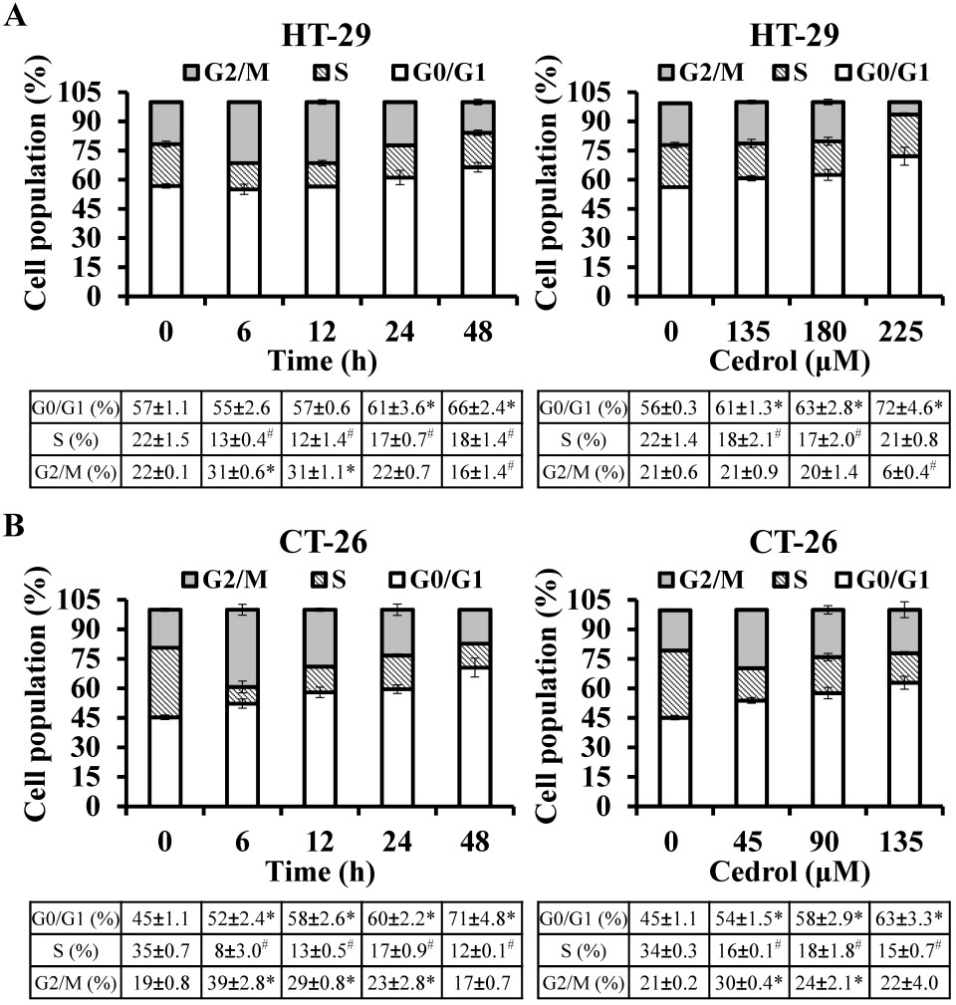
** Flow cytometry detection of cell cycle progression.** HT-29 **(A)** and CT-26 **(B)** cells were treated with cedrol for time-course and dose-response experiments, respectively. At the end of the treatment, the cells were harvested, stained with PI, and subjected to cell cycle analysis using FACScan and FlowJo software. Data are expressed as the mean ± SD of triplicate determinations. *p < 0.05, compared to the control, with a significant increase. ^#^*p* < 0.05, compared to the control, with a significant decrease.

To determine whether cedrol-induced proliferation inhibition of CRC cells was mediated via the regulation of cell cycle progression, flow cytometry analysis with PI staining was performed. The precise assessment of cellular DNA content by flow cytometry classifies the various phases of the cell cycle, including G_0_/G_1_, S, and G_2_/M phases. As shown in Figure 2A and B, compared with the control group, HT-29 and CT-26 cells were arrested at the G_0_/G_1_ in a time- and dose-dependent manner; the percentage of HT-29 cells in the G_0_/G_1_ phase increased from 56.84% to 66.45% (time-course) and from 56.23% to 72.12% (dosage) respectively, and the percentage of CT-26 cells in the G_0_/G_1_ phase increased from 45.32% to 70.58% (time-course) and from 45.15 to 62.89 (dosage), respectively.

**Figure 3:**
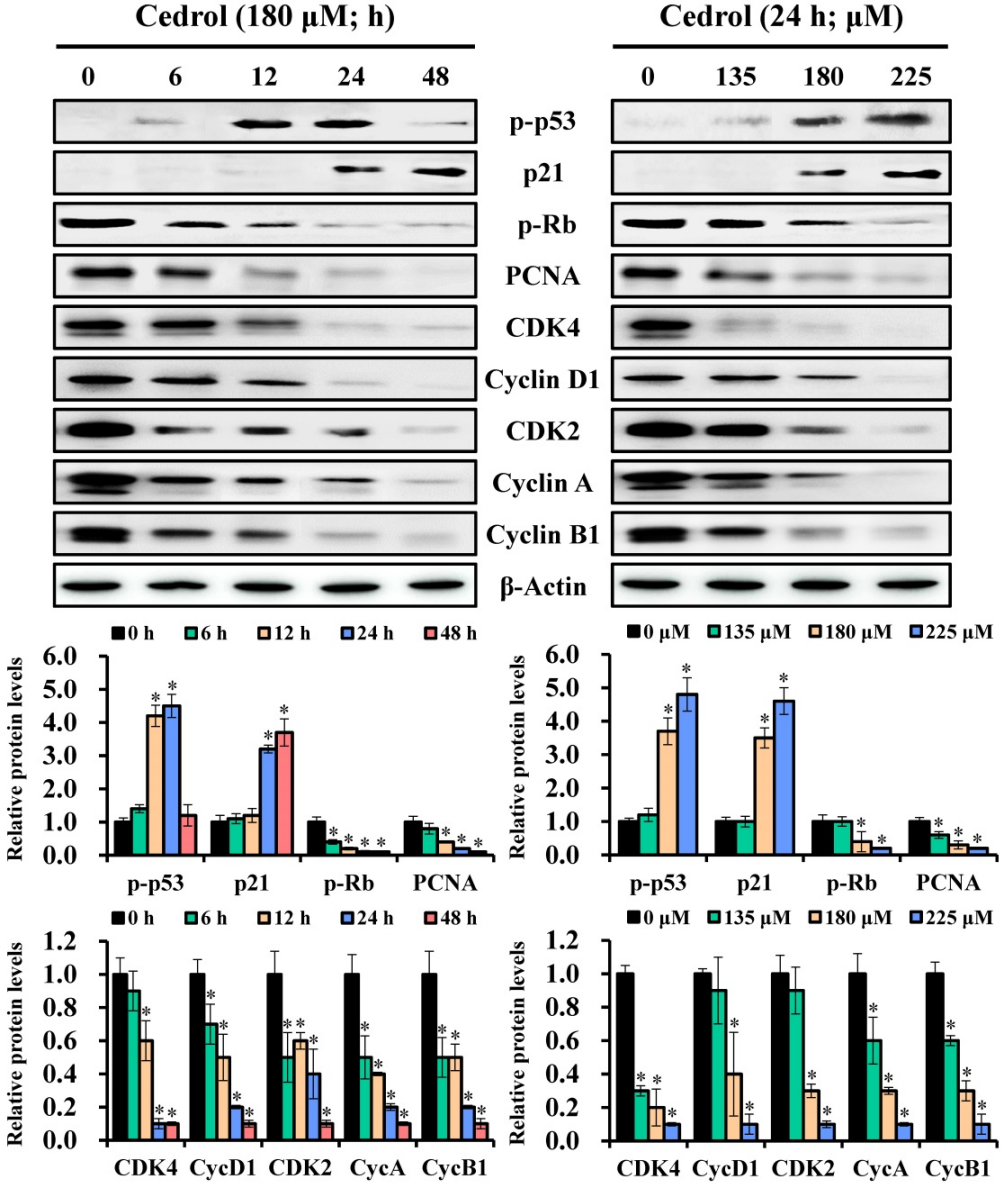
** Analysis of the time-course or dose-effect of cedrol on expression of cell cycle-related proteins.** HT-29 cells were treated with cedrol (180 µM) for 0, 6, 12, 24, and 48 h; cedrol (0, 135, 180, and 225 µM) for 24 h, and then collected and lysed in RIPA buffer. Cell lysate was subjected to SDS-polyacrylamide gels and analyzed using western blotting. The density of the integrated band was determined using ImageJ software, and β-actin was used as an internal control. Data were expressed as the mean ± SD of three separate experiments. *p < 0.05, compared to control. PCNA, proliferating cell nuclear antigen; Rb, retinoblastoma protein; CDK, cyclin-dependent kinase.

To understand the possible molecular mechanisms related to the growth arrest of CRC cells induced by cedrol, the regulatory proteins of the cell cycle were detected by western blot analysis. Immunoblot results showed that the expression levels of p-p53 and p21 in cedrol-treated cells were increased, whereas those of p-Rb and PCNA were reduced in a time- and dose-dependent manner (Figure 3). Concurrently, the cells treated with cedrol showed a decrease in the G_0_/G_1_ phase-associated proteins CDK4 and cyclin D1 and regulators of other phases such as CDK2, cyclin A, and cyclin B1. These findings suggested that cedrol upregulated p-p53/p21 expression and downregulated CDK4/cyclin D1 expression and contributed to the suppression of CRC cells by cedrol-induced G_0_/G_1_ arrest.

### Cedrol Triggered Extrinsic and Intrinsic Apoptosis Pathways

**Figure 4:**
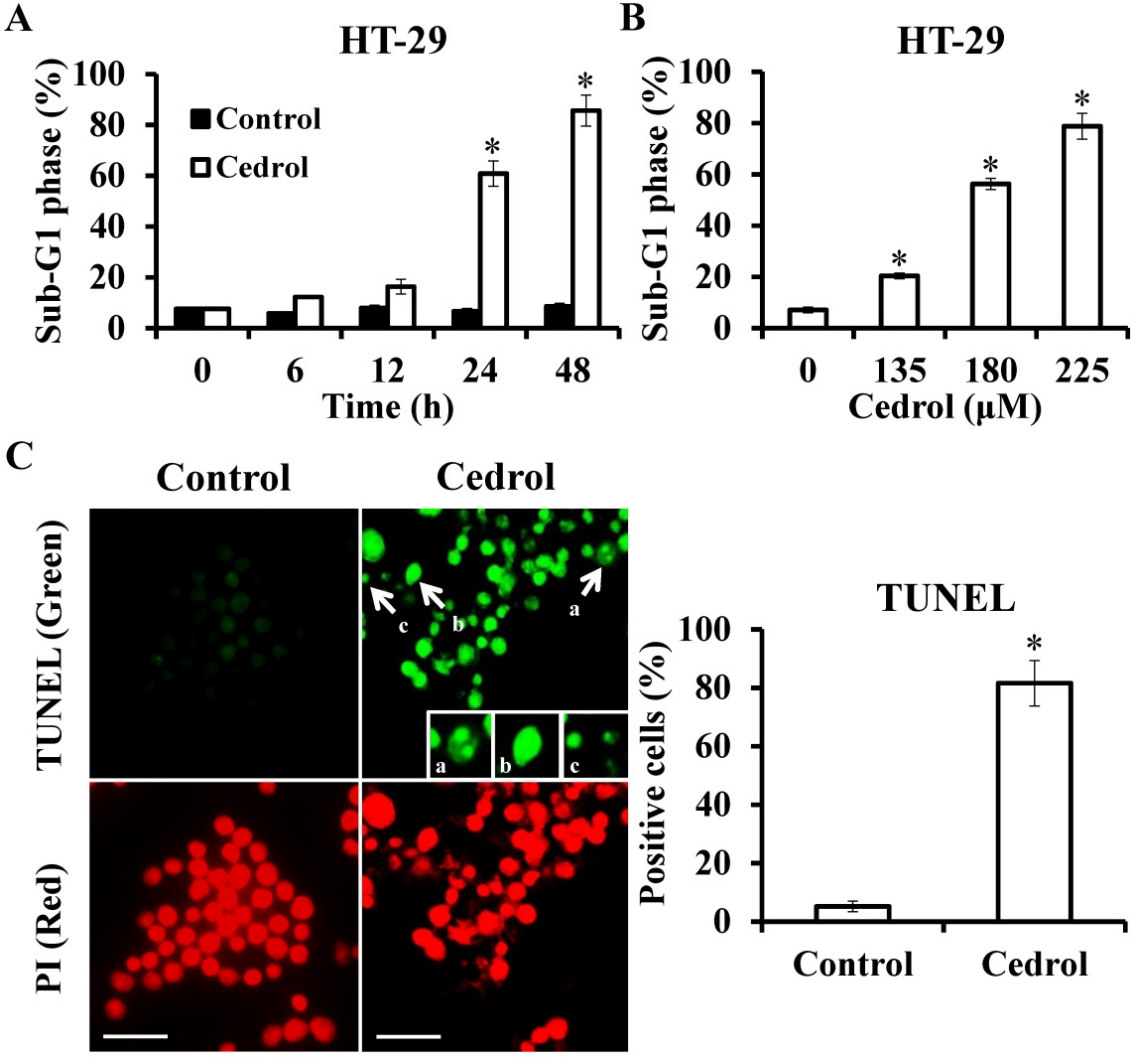
** Detection of apoptosis in cedrol-treated cells using TUNEL staining. (A, B)** HT-29 cells were treated with cedrol, and the percentage of cells at the sub-G_1_ phase by flow cytometry was analyzed. **(C)** HT-29 cells were treated with 180 µM of cedrol for 48 h, followed by the detection of apoptosis using a TUNEL assay kit. The green fluorescence indicated TUNEL-positive cells, and red fluorescence (PI counterstain) indicated the site of a nucleus, visualized by fluorescence microscopy. a, chromatin condensation; b, DNA fragmentation; c, apoptotic body. Scale bar = 25 µm. The results were shown as the mean ± SD. *p < 0.05, compared to control.

Flow cytometry analysis demonstrated that treatment of the cells with cedrol resulted in the accumulation of cells in the sub-G_1_ phase in a time- and dose-dependent manner (Figure 4A and B). Up to 80% of the cells were in the sub-G_1_ phase after treatment with 180 μM cedrol for 48 h, as indicated by the fluorescent signal. To confirm whether this accumulation was a result of apoptosis, HT-29 cells were treated with cedrol, stained with TUNEL, and observed using fluorescence microscopy. Apoptotic cells usually exhibited characteristic morphologic features, such as chromatin condensation, DNA fragmentation, and apoptotic bodies, which could be observed as green fluorescent signals after TUNEL staining. As shown in Figure 4C, TUNEL-positive cells were significantly increased from 5.20 ± 1.79% to 81.6 ± 7.77% and exhibited typical apoptotic morphology as described above.

**Figure 5:**
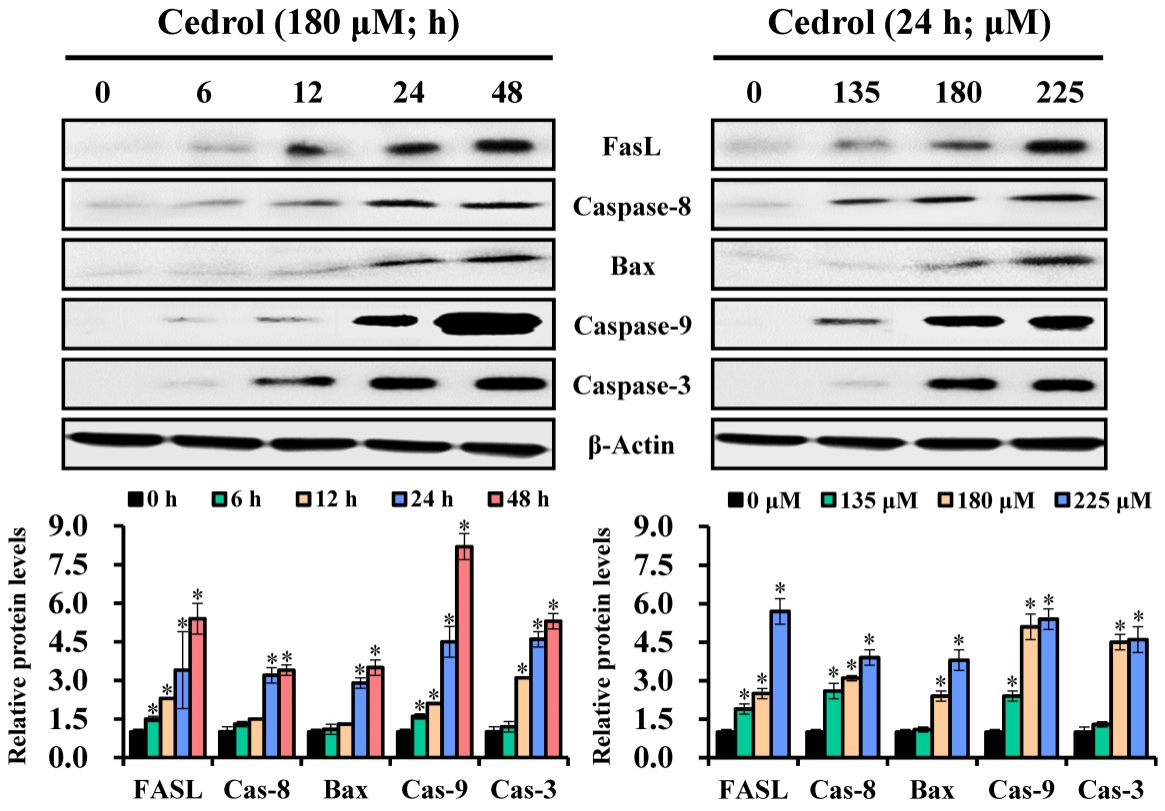
** The effect of cedrol on activation of caspase apoptosis cascade.** HT-29 cells were treated with 180 µM cedrol for 0-48 h or cedrol at the indicated concentrations (0, 135, 180, and 225 µM) for 24 h. Total cell lysates were separated on SDS-polyacrylamide gels and blotted onto PVDF membranes. The membranes were probed with FasL, caspase-8, Bax, caspase-9, caspase-3, and β-actin antibodies. The expression of proteins was detected using an electrochemiluminescence system and semi-quantified using ImageJ software. β-actin was used as an internal control. Data are shown as mean ± SD from three separate experiments. *p < 0.05, compared to the control. FasL, Fas ligand.

Subsequently, to identify the cell-death pathways induced by cedrol, western blot analysis was performed to detect the key proteins involved in apoptotic cell death involving the extrinsic and intrinsic apoptosis pathways. The data presented in Figure 5 demonstrate that cedrol treatment significantly increased the expression levels of FasL/cleaved-caspase-8, Bax/cleaved-caspase-9, and cleaved-caspase-3 in a time- and dose-dependent manner. These results clearly showed that cedrol induced apoptosis in CRC cells via the activation of both extrinsic and intrinsic apoptosis pathways.

### Combination of Cedrol and 5-FU Synergistically Inhibited Growth of CRC Cells

**Figure 6:**
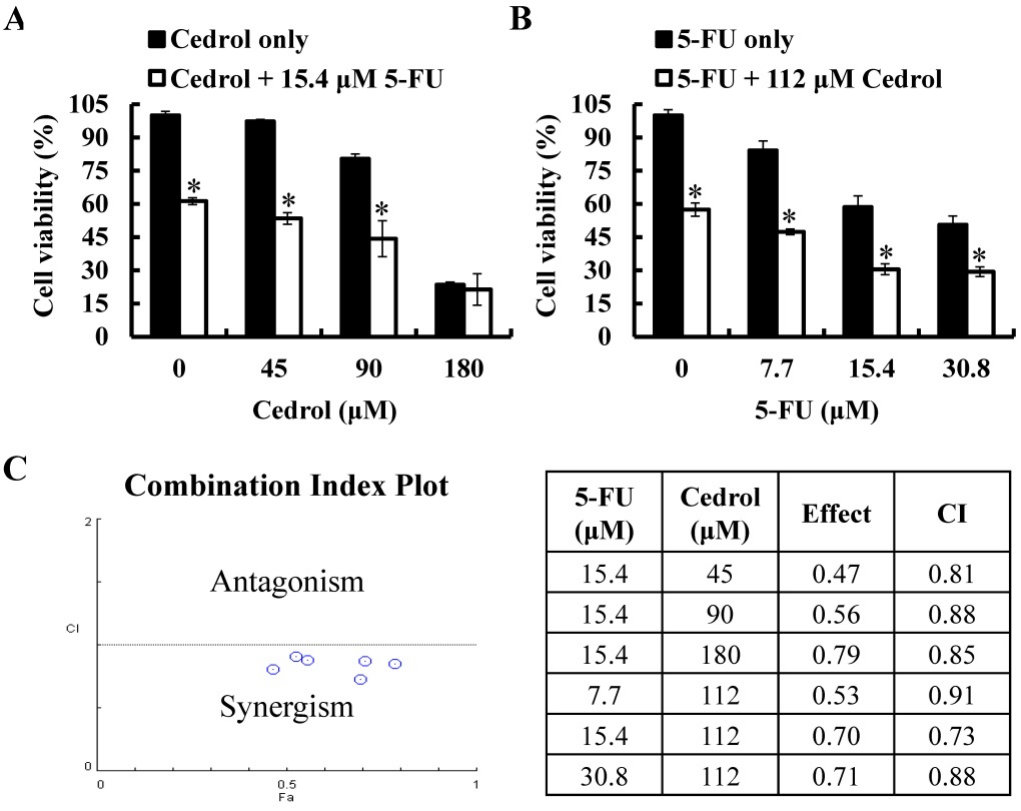
** The effect of cedrol combined with 5-FU on growth of CRC cells.** HT-29 cells were treated with **(A)** cedrol (0, 45, 90, and 180 µM) combined with 15.4 µM 5-FU; **(B)** 5-FU (0, 7.7, 15.4, and 30.8 µM) combined with 112 µM cedrol for 48 h, after which cell viability was measured using the MTT assay. The results are presented as mean ± SD. *p < 0.05, compared to control or 5-FU alone. **(C)** The combination index (CI) of the combination treatment of cedrol and 5-FU in HT-29 cells was determined using CompuSyn software to evaluate synergism (CI < 1), additive effects (CI = 1), and antagonism (CI > 1).

To investigate the synergistic effect of cedrol on the anti-tumor proliferation effect of 5-FU, HT-29 cells were treated with cedrol (45, 90, and 180 μM) combined with 5-FU (15.4 μM, IC_40_) or 5-FU (7.7, 15.4, and 30.8 μM) combined with cedrol (112 μM, IC_40_) for 48 h, followed by the detection of cell viability using MTT assay. As shown in Figure 6A and B, after co-treatment with cedrol and 5-FU, the survival of HT-29 cells was significantly reduced in comparison with cedrol or 5-FU treatment alone. The combination index (CI) was used to determine the effect of drug-drug interactions and offered a quantitative definition of the additive effect (CI=1), synergism (CI < 1), and antagonism (CI > 1), calculated by the computational model software CompuSyn based on the Chou-Talalay method. All CI values of the different dose combinations were below 1 and ranged from 0.73 to 0.91 (Figure 6C). These results indicate a synergistic effect in the growth inhibitory activity of cedrol and 5-FU in CRC cells.

### Cedrol Suppressed CT-26 Tumor Growth *in vivo*

**Figure 7:**
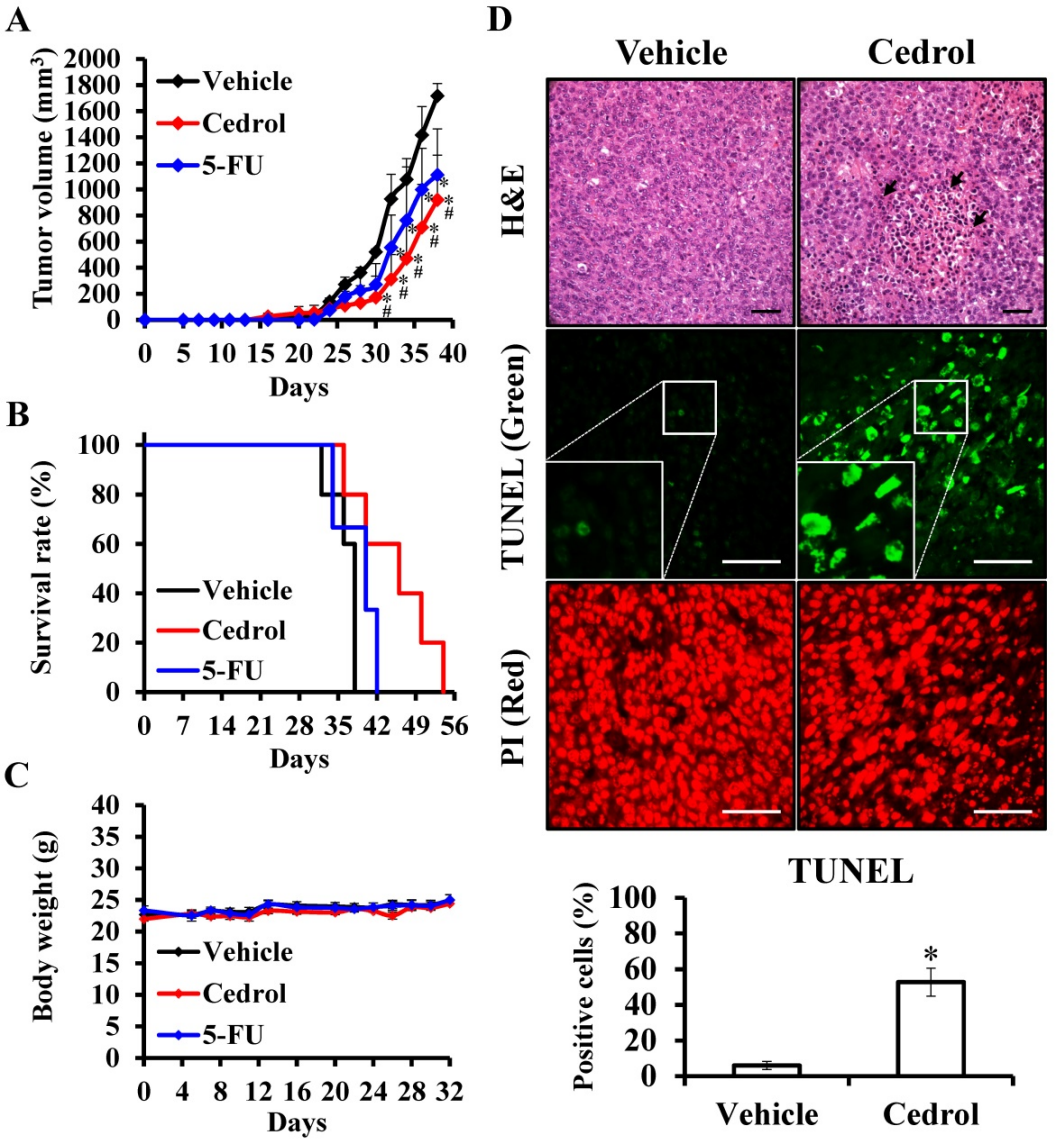
** The effect of cedrol on tumor suppression of CRC *in vivo.*** Mice were subcutaneously injected with 1 × 10^6^ CT-26 cells and randomly divided into three groups: vehicle (n = 5), control (n = 5) and 5-FU (n = 3). After 7 days, the mice were treated once every 2 days by subcutaneous injection of 150 mg/kg cedrol for 10 times, or 3 times a week by intraperitoneal injection of 25 mg/kg for 21 days. During the experiments, the tumor volume **(A)**, survival rate **(B)**, and body weight **(C)** were recorded. **(D)** Paraffin-embedded tissue sections were prepared from the tumors of treated and untreated mice, and H&E and TUNEL staining were performed. TUNEL-positive (green) cells were apoptotic cells; nuclei were labeled with propidium iodide (PI) stain (red) and analyzed using fluorescence microscopy. Arrowheads indicate nucleolysis. All results are presented as the mean ± SEM. *p < 0.05, compared with vehicle. Scale bar, 50 µm.

To examine the *in vivo* potential therapeutic effects of cedrol, mouse-harbouring CRC cell line-derived tumor models were used. Seven days after subcutaneous injection of murine CT-26 colon carcinoma cells into the right dorsal flank of Balb/c mice, mice were treated subcutaneously with 150 mg/kg of cedrol or solvent alone (vehicle group) once daily 10 times. From day 28 onwards, the differences in tumor volumes between the two groups were highly significant, and the average tumor volume of the treated mice was reduced by 46.38% compared with those in the vehicle group at day 38 (Figure 7A). In comparison with the vehicle group, the lifespan of cedrol-treated mice was significantly prolonged from 38 to 54 days (Figure 7B). Although the clinical drug 5-FU significantly suppressed tumor growth, the inhibitory effect was not as good as that in the cedrol group. During the complete study period, the mean body weight of the mice remained virtually unaltered in the three groups, and no significant difference was observed (Figure 7C).

To study the underlying mechanism of cedrol *in vivo*, histological sections of tumor specimens obtained from treated and untreated tumor-bearing mice were stained using different techniques (Figure 7D). H&E staining showed that the density of tumor cells was greatly decreased compared to that in the vehicle group, and nucleolysis was observed after cedrol treatment. Additionally, an 8.8-fold increase in apoptotic cells was detected in TUNEL-stained tumor sections of cedrol-treated mice compared to that in the vehicle group. These findings demonstrated that cedrol suppressed CRC tumor growth *in vivo* by inducing cell apoptosis, consistent with the results of the *in vitro* study.

## Discussion

Various strategies have been developed for the treatment of CRC, including surgery, chemotherapy, radiotherapy, targeted therapy, and immunotherapy 3. Chemotherapy is usually used before or after surgical resection to prevent metastasis and recurrence of cancer; however, it is restricted by systemic toxicity, including thrombocytopenia, leukopenia, anemia, diarrhea, and other adverse reactions 5, 11. In addition, adjuvant chemotherapy cannot be completely controlled, and relapse occurs in approximately 30% of patients with stage I-III disease and 65% of post-stage IV patients 34. Therefore, there is an urgent need to develop new drugs with high efficiency and low toxicity for CRC treatment. Recent studies of cancer treatment strategies indicate that the regulation of cell division or apoptosis by targeting specific biomarkers selectively affects the growth of cancer cells and shows less toxicity to normal cells 15. In this study, we chose HT-29 (human colorectal adenocarcinoma) and CT-26 (mouse colorectal adenocarcinoma) as colon cancer cell lines and used them in *in vitro* and *in vivo* studies, respectively. The results demonstrated that cedrol inhibited the growth of CRC cells via induction of cell cycle arrest and apoptosis and demonstrated higher selectivity toward CRC cells compared to normal cells. Cedrol also suppressed tumor progression in tumor-bearing mice with no observed adverse effects. Based on our findings, cedrol is a potential candidate as an anticancer drug for CRC treatment.

Natural compounds are candidates for targeted therapy of various cancers, with fewer side effects 21. Cedrol, derived from *Cedrus atlantica*, is a natural compound with a wide range of pharmacological activities, such as antimicrobial, anti-inflammatory, analgesic, and anti-anxiety activities, including the promotion of hair growth 24-28, and has been shown to be effective in the treatment of cancer. Cedrol reduces cell viability in human amelanotic melanoma C32 cells and renal adenocarcinoma ACHN cells in a dose-dependent manner, with an IC_50_ of 199.49 and 184.65 µM at 48 h, respectively 35. It also exerts inhibitory effects in human lung cancer A549 cells (IC_50_ values were 31.88, 14.53, and 5.04 µM at 24, 48, and 72 h) and mediates apoptosis and autophagy through the regulation the PI3K/Akt signaling pathway, generation of reactive oxygen species, and loss of mitochondrial transmembrane potential 29. In addition, cedrol activates intrinsic apoptosis, suppresses the AKT/ERK/mTOR and NF-κB signaling pathways, and chemosensitizes cancer cells through lipid raft destabilization in human leukemia K562 and colon cancer HT-29 cells, with an IC_50_ of 179.52 and 185.50 µM at 48 h, respectively 32. More recently, cedrol was reported to suppress the growth of glioblastoma and triggered apoptosis by the induction of DNA damage and targeting the androgen receptor (IC_50_ values of glioblastoma cells were 77.17-141.88 µM at 48 h) 30. Although many studies have suggested the anti-cancer ability of cedrol and its ability to inhibit proliferation and induce apoptosis, its therapeutic effect and possible mechanism *in vivo* in CRC remain unknown. In the present study, our findings showed that the IC_50_ values of cedrol were 138.91 μM and 92.46 μM at 48 h in HT-29 and CT-26 cells, respectively. Moreover, cedrol suppressed CRC growth by inducing apoptosis both *in vitro* and *in vivo*. These results were consistent with those of previous studies and further proved the anti-CRC effects of cedrol *in vivo*, providing new evidence for the drug development of cedrol in the future.

The cell cycle is a complex event in which one cell replicates its DNA and divides to form two daughter cells, and it is commonly deregulated in cancer cells, resulting in uncontrolled proliferation 36. Cell cycle regulation involves various regulatory proteins, such as CDKs/cyclins, tumor suppressor genes, interphase oncogenes, and mitotic checkpoint proteins. CDKs/cyclins are a family of heterodimeric kinases that play a key role in regulating cell cycle progression in different phases, including the G_0_/G_1_ phase (CDK4/6 and cyclin D1), S phase (CDK2 and cyclin E), G_2_/M phase (CDK1/2 and cyclin A), and cyclin B1 37. According to reports, the dysregulated CDKs in cancer cells are correlated with unscheduled proliferation and instability of genome or chromosome 36. Thus, the regulation of the cell cycle or relative regulators is important for controlling the growth of cancer cells. In this study, G_0_/G_1_ phase accumulation in HT-29 and CT-26 cells was upregulated by cedrol treatment in a time- and dose-dependent manner. Cedrol treatment resulted in increased expression of p-p53 and p21 proteins, which are tumor suppression proteins that block cell cycle progression. Moreover, cedrol downregulated the expression of CDK4, cyclin D1, CDK2, cyclin A, and cyclin B1 in a time- and dose-dependent manner. Although the cell cycle was mainly arrested at the G_0_/G_1_ phase, overall, the expression of cell cycle-related proteins was reduced following exposure to cedrol. The results of the present study demonstrated that cedrol suppressed the proliferation of CRC cells by regulating the expression of p-p53/p21 and CDKs/cyclins to arrest the cell cycle.

Apoptosis is an orderly and tightly programmed process of cell death and is regarded as a promising strategy for the development of chemotherapy to treat different types of cancer 15. In the present study, cells at the sub-G_1_ phase of the cell cycle accumulated after cedrol treatment. In addition, the induction of apoptosis was studied using double staining (TUNEL and PI), which showed increased percentages of apoptotic cells and typical morphology, such as chromatin condensation, DNA fragmentation, and apoptotic bodies, in cedrol-treated cells. Caspase activation is a crucial step in the induction of apoptosis and is divided into intrinsic and extrinsic pathways 19. The binding of the Fas ligand triggers Fas clustering and its binding to Fas-associated protein with the death domain, which recruits caspase-8 and caspase-10 to form the death-inducing signaling complex, resulting in activation of the intrinsic apoptotic pathway 38. Bax/Bcl-xL is considered an important parameter, and increasing the ratio promotes the activation of cytochrome C and pro-apoptotic proteins to initiate intrinsic apoptosis. 39. Thereafter, the cleaved caspase-8 and caspase-9 (active form) induce the activation of caspase-3 and downstream proteins, which subsequently leads to the cleavage of short amino acid sequences, causing cell death 20. In this study, cedrol significantly elevated the levels of extrinsic (FasL/caspase-8) and intrinsic (Bax/caspase-9) apoptotic pathway proteins, followed by activation of caspase-3. These results indicate that cedrol induced apoptosis via caspase-dependent pathways in CRC HT-29 cells.

In the last few decades, 5-FU has been widely used as a chemotherapeutic agent for CRC treatment. However, its clinical efficacy has been limited owing to the development of drug resistance and occurrence of systemic adverse effects after long-term treatment with high-dose 5-FU 40. Moreover, the response of patients to 5-FU treatment is dependent on the p53 status of cancer cells, with mutant p53 exhibiting higher drug resistance 41. One strategy to improve drug resistance and drug-induced toxicity is to combine classic therapy with adjuvant treatment to reduce the dose of chemotherapeutic drugs and replace them with other natural drugs that are less toxic to normal cells. Combination treatment with reduced doses of 5-FU and compounds from natural sources reportedly enhances the growth inhibition of cancer cells compared to high-dose 5-FU alone 42. Thus, we investigated the effect of cedrol as an adjuvant in combination with 5-FU in HT-29 cells with mutated TP53. Our data showed that the combination of cedrol and 5-FU had synergistic effects, which enhanced the reduction in the proliferation of HT-29 cells. Cedrol combined with the clinical drug temozolomide also exhibits synergistic suppression in glioblastoma by downregulation of AKT/mTOR signaling and chemosensitizes human leukemia K562 and CRC HT-29 cells by destabilizing plasma membrane lipid rafts 30-32. Therefore, the combination of cedrol and 5-FU may be a promising strategy for overcoming the limitations of 5-FU in CRC treatment.

## Conclusion

In this study, we provide evidence for the significant anti-cancer activity of cedrol in CRC both *in vitro* and *in vivo*. Cedrol downregulated the expression of CDK4/cyclin D1 proteins and induced cell cycle arrest at the G_0/_G_1_ phase. In addition, activation of extrinsic (FasL/caspase-8) and intrinsic (Bax/caspase-9) apoptotic pathways led to the induction of apoptosis by cedrol treatment. The combination of cedrol and 5-FU exhibited synergistic effects on CRC cell growth. To the best of our knowledge, this study is the first to demonstrate *in vivo* inhibitory effects of cedrol against CRC and its underlying anti-cancer mechanism. The findings of this study suggest that cedrol could potentially be useful as an effective therapeutic agent or adjuvant for CRC treatment.

## Figures and Tables

**Table 1:** IC_50_ values of cedrol and 5-FU against HT-29 and CT-26 cells

Cell line	Tumor type	Cedrol	5-FU
HT-29	human colorectal adenocarcinoma	138.91 ±17.81^#^	47.89 ±12.22
CT-26	mouse colorectal carcinoma	92.46 ± 4.09^#^	< 24.02
SVEC	mouse vascular endothelial cell	202.19 ± 4.27	< 24.02
MDCK	canine kidney epithelial cell	281.60 ± 5.17	93.79 ±0.69

The half-maximal inhibitory concentration (IC_50_) values were calculated from dose-response curves of cedrol or 5-FU at 48 h and showed as means ± SD (μM) from at least three independent experiments performed in triplicate. ^#^*P*<0.05 between CRC and normal cells.

**Table 2:** The selective index between CRC and normal cells

Normal cells (IC_50_) / Tumor cells (IC_50_)	Cedrol	5-FU
SVEC/HT-29	1.46	<0.50
MDCK/HT-29	2.03	1.96
SVEC/CT-26	2.19	N.C.
MDCK/CT-26	3.05	>3.90

Selectivity index (SI) was calculated as the IC_50_ value in the normal cell line divided by the IC_50_ value in the CRC cell line. The higher the SI value, the greater the selectivity of the cedrol toward the CRC cells. N.C., not calculated.
